# Optimization and Validation of High-Performance Chromatographic Condition for Simultaneous Determination of Adapalene and Benzoyl Peroxide by Response Surface Methodology

**DOI:** 10.1371/journal.pone.0120171

**Published:** 2015-03-20

**Authors:** Yi-Cheng Chen, Pi-Ju Tsai, Yaw-Bin Huang, Pao-Chu Wu

**Affiliations:** 1 School of Pharmacy, College of Pharmacy, Kaohsiung Medical University, Kaohsiung, Taiwan, Republic of China; 2 Department of Business Administration, I-Shou University, Kaohsiung, Taiwan, Republic of China; Irvine, UNITED STATES

## Abstract

The aim of this study was to develop a simple and reliable high-performance chromatographic (HPLC) method for simultaneous analysis of adapalene and benzoyl peroxide in pharmaceutical formulation by response surface methodology (RSM). An optimized mobile phase composed of acetonitrile, tetrahydrofuran and water containing 0.1% acetic acid at a ratio of 25:50:25 by volume was successfully predicted by using RSM. An isocratic separation was achieved by using the condition. Furthermore, the analytical method was validated in terms of specificity, linearity, accuracy and precision in a range of 80% to 120% of the expected concentration. Finally, the method was successfully applied to the analysis of a commercial product.

## Introduction

Acne vulgaris is a common chronic skin disease characterized by comedones, papules, and pustules and is associated with negative sequelae including scarring and pigmentation, which causes a high degree of psychosocial suffering [1–3]. It affects about 85% of individuals between the ages of 12 to 24 years [4,5]. Topical antibiotics, keratolytics, and retinoids are the main first-line treatment options for comedogenic and mild inflammatory acne. Combination therapy is often used in many clinical settings in order to target different pathogenic factors [4,6–8].

**Fig 1:**
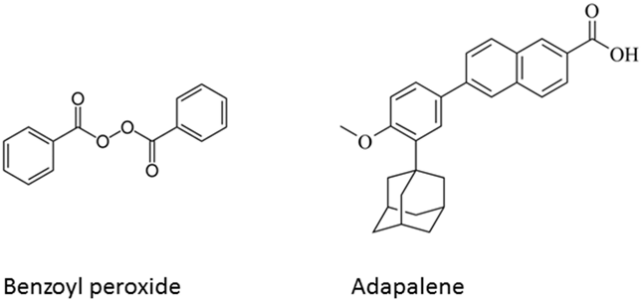
Chemical Structure of adapalene and benzoyl peroxide.

Adapalene, (6-[3-(1-adamantyl)-4-methoxyphenyl]-2- naphthoic acid) (Fig. 1) is a synthetic naphthoic acid derivative, third-generation retinoid with slightly better efficacy and light stability [9,10]. Retinoids can reverse the abnormal follicular desquamation and have anti-inflammatory effects. Topical retinoids are used in the treatment of mild-to-moderate acne and are also used (off-label) to treat keratosis pilaris as well as other skin conditions. In comparison with other topical retinoids, adapalene shows lower incidence of side effects and exhibits better tolerability profile, hence is clinically above the rest in the treatment of acne vulgaris [10–12]. Benzoyl peroxide (Fig. 1) can increase skin turnover, clearing pores and reducing the bacterial count. It also has anticomedogenic and mild keratolytic effect [8,9,13,14]. Hence, benzoyl peroxide is one of the most widely prescribed drugs in acne therapy. A combination of adapalene and benzoyl peroxide shows good stability and a good clinical tolerance [15], and has also been found to have better tolerability than BPO monotherapy in terms of cutaneous tolerability [9]. Thus, the first topical acne preparations combining adapalene 0.1% and benzoyl peroxide 2.5% were approved by the US Food and Drug Administration in 2008 [9,16].

There are some reported analytical methods for the determination of adapalene [15,17,18] or benzoyl peroxide alone [19–21] by high-performance liquid chromatography (HPLC) and capillary electrophoresis in bulk or in pharmaceutical dosage form. The purpose of this study was to develop and validate a simple HPLC method for simultaneous analysis of adapalene and benzoyl peroxide in pharmaceutical product. In order to obtain an optimal analytical condition in a short time period and minimum trails, a statistical optimization tool based on response surface methodology (RSM) with a constrained mixture design [22,23] was used. The optimization procedure involved systematic design to minimize the number of trails, and analysis the response surfaces to realize the influence of independent factors and to obtain the optimal condition. In this present study, RSM was used to search for the appropriate mobile phase for the analysis method. Then, the analytical method was validated for specificity, linearity, accuracy, precision and robustness according to the guidelines for assay methods of the International Conference on Harmonisation of Technical Requirements for Registration of Pharmaceuticals for Human Use (ICH) [24] and applied to the analysis of a commercial product.

## Materials and Methods

### 2.1. Materials

Adapalene with purity of 98% was purchased from Glenmark Generics Limited (Mumbai, India). Benzoyl peroxide with purity of 75% was purchased from Yuh Tzong Enterprise Ltd (Kaohsiung, Taiwan). Acetonitrile and methanol were obtained from Echo Chemical Co. (Miaoli, Taiwan). Tetrahydrofuran was obtained from J.T.Baker (Pennsylvania, U.S.A.). Purified water was prepared using Milli-Q (Millipore, MA, U.S.A.) All other chemicals and reagents used in the experiment were analytical grade. The commercial gel product (Epiduo) containing 0.1% adapalene and 2.5% benzoyl peroxide was purchased from Galderma (Levallois-Perret, France).

### 2.2. Standard solution preparation

The stock solutions of adapalene and benzoyl peroxide were prepared by simultaneously dissolving 4 mg adapalene and 100 mg benzoyl peroxide in 50 ml methanol. The standard solution was prepared by diluting the stock solution with methanol to achieve a final nominal concentration of 40 μg/ml adapalene and 1 mg/ml benzoyl peroxide.

### 2.3. Instrumentation and chromatographic conditions

The HPLC system used consisted of a chromatographic pump (L-7100), autosampler (L-2200), and an UV detector (L-4000H) (Hitachi, Japan). The LiChrosorb C_18_ (250×4.6mm, particle size 5 μm) column and guard column 4–4 packed with LiChrosper 100 RP-18 (Merck, Darmstadt, Germany) were used. The analytical column and the guard column were operated at ambient temperature. The injection volume was 20 μL. Detection was performed at 270 nm. The flow rate was set at 1.0 ml/min.

To obtain an optimal mobile phase for the analytical method, the statistical optimization tool based on a RSM with a constrained mixture design [22,23] was used. According to the USP 36 [18], the mobile phase contains acetonitrile, tetrahydrofuran, trifluoroacetic acid, and water at ratios of 21: 16: 0.01: 13 for determination of adapalene. USP 34 [21] reported a gradient mobile phase consists of acetonitrile and 0.1% glacial acetic aqueous solution ranged from 82/12 to 60/40 in 30 mins for benzoyl peroxide analysis. Hence, tetrahydrofuran (X_1_), acetonitrile (X_2_) and water containing 0.1% acetic acid (X_3_) was chosen as the variable factors of mobile phase in this study. The levels of X_1_, X_2_ and X_3_ were set from 10–30, 50–70 and 15–25%, respectively. Based on the constrained mixture model provided by Design-Expert software, ten model mobile phase compositions were arranged randomly. Two replicated batches of R02 vs R10 and R03 vs R09 were used to estimate the reproducibility by lack of fit. The compositions of mobile phase are listed in Table 1. The tailing factor (TF), retention time (RT), theoretical plates (TP) and resolution of adapalene and benzyl peroxide (AB/R) were used to evaluate the availability of mobile phases.

**Table 1:** The composition and analysis parameters of the HPLC method.

	Code value			Adapalene			Benzoyl peroxide			
	X_1_	X_2_	X_3_	TF	RT	TP	TF	RT	TP	AB/R
R01	0.4	0.4	0.2	1.35	12.3	8649	1.12	3.6	8630	22
R02	0.8	0.2	0.0	1.31	5.9	8045	1.16	2.9	7967	13
R03	0.2	0.8	0.0	1.45	10.1	8002	1.17	3.2	8317	20
R04	0.0	0.8	0.2	1.49	17.4	8791	1.13	3.9	8886	25
R05	0.0	0.6	0.4	1.43	25.3	9131	1.12	4.6	9732	28
R06	0.6	0.0	0.4	1.27	12.9	8671	1.16	3.8	8939	22
R07	0.2	0.6	0.2	1.41	14.3	8487	1.14	3.7	8719	23
R08	0.2	0.4	0.4	1.34	20.4	9217	1.13	4.3	9376	27
R09	0.2	0.8	0.0	1.45	9.9	8006	1.16	3.2	8204	20
R10	0.8	0.2	0.0	1.31	5.7	7643	1.20	2.8	7816	13

X_1_: tetrahydrofuran, X_2_: acetonitrile, X_3_: water containing 0.1% acetic acid.

TF: tailing factor, RT: retention time, TP: theoretical plates, AB/R: resolution between adapalene and benzoyl peroxide.

### 2.4. Method validation

The HPLC method was validated for specificity, linearity, accuracy, precision and robustness according to the guidelines for assay methods of the International Conference on Harmonisation of Technical Requirements for Registration of Pharmaceuticals for Human Use (ICH) [24].

#### As Specificity

The standard solution was used to assess the retention times. Blank chromatogram was recorded to check for interfering matrix peaks. Finally, chromatogram of real sample solution was reviewed for critical peak separations. Whenever appropriate, chromatographic resolution was calculated.

#### As Linearity

To evaluate the linearity of the method, five standard solutions (80%~120% of nominal concentration) were prepared. Each concentration level was analyzed in triplicate. The linearity study was performed over two days. The peak areas were plotted versus the respective concentrations, and a linear regression analysis was used to obtain the equation and correlation coefficients. The limit of detection was the lowest amount that could be detected S/N >3.

#### As Precision/Accuracy/Recovery

The intra-day precision and accuracy were evaluated by measuring five replicates of the samples at three concentrations levels (80, 100 and 120% of nominal concentration) on the same day, whereas the inter-day precision and accuracy were estimated using three validation batches on three consecutive days. The precision was calculated as the relative standard deviation (RSD%), and the accuracy was defined as the relative error (E%). Recovery was calculated as the ratio of the amount of drug from spiked gel to the amount of analyte added.

#### As Robustness

The robustness was performed by modifying the experimental conditions. The chromatographic parameters including peak area, retention time, theoretical plates, and tailing factor were evaluated for different detected wavelengths (268 and 272 nm) and tetrahydrofuran concentrations (24 and 26%) and flow rate (0.9 and 1.1 mL/min).

### 2.5. Application of the analytical method in pharmaceutical gel

Accurately weighed amounts of pharmaceutical gel 2 g corresponding to 2 mg of adapalene and 50 mg of benzoyl peroxide was added into a 50 ml-volumetric flask. About 40 ml of methanol was added to this volumetric flask and sonicated in an ultrasonic bath for 15 min with intermittent shaking, diluted to volume with methanol, and mixed well. The sample solution of concentration (nominal concentration) was 40 μg/ml of adapalene and 1 mg/ml of benzoyl peroxide. Before being analyzed by HPLC, a portion of sample solution was centrifuged in tube caps at 12000 rpm for 10 min and filtered through a 0.45 μm Durapore membrane filter. A solution of blank gel containing the same excipients with commercial gel treated by the same procedure was used as a blank sample in the spectrophotometric method.

## Results and Discussion

### 3.1. Optimization of mobile phase

The parameters of analysis capability of HPLC method in different mobile phase compositions are listed in Table 1. The TF, RT and TP were 1.27–1.49, 5.9–25.3 min and 7643–9217 for adapalene, and 1.12–1.20, 2.8–4.6 min and 7816–9732 for benzoyl peroxide respectively. The AB/R ranged from 13 to 22. These results indicated that the composition of the mobile phase had significant effect on the analysis capability of this analytical method.

**Fig 2:**
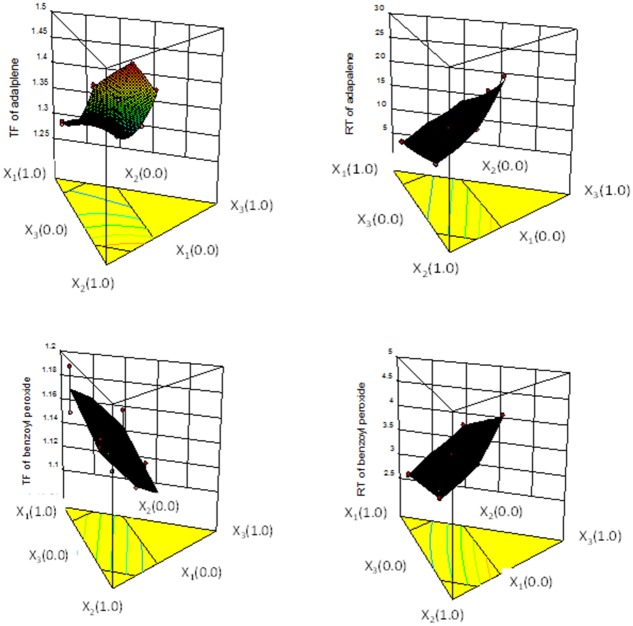
The three-dimensional contour diagrams illustrating the effect of tetrahydrofuran (X_1_), acetonitrile (X_2_) and water containing 0.1% acetic acid (X_3_) on the retention time (RT) and tailing factor (TF).

To determine the effect of composition of mobile phase on the analysis capability, the values of TF and RT of adapalene and benzoyl peroxide were set as responses. The TP and AB/R in all conditions were far greater than the requirement of 4000–6000 and 1.5, so does not assess in this study. These responses and three independent variables (X_1_, X_2_ and X_3_) were statistically analyzed based on the response-surface methodology using a computer program (Design-Expert software) [22,25]. The results of multiple regression analysis for all parameters studied are summarized in Table 2. The probability (*p*-value) of the model was less than 0.05, indicating that the fitted model could describe the relationship between the dependent and independent variables. The *p*-value for the lack of fit for all models were greater than 0.05, suggesting absence of any lack of fit of the models, and that also strengthened the reliability of the models. The values of the coefficients X_1_, X_2_ and X_3_ were related to the effect of these variables on the response. The result showed that the aqueous phase (X_3_) had more influence degree on the RT, whereas organic phases (X_1_ and X_2_) had stronger effect on TF for both analytes. The phenomena might be attribute to the reverse phase column was used for both hydrophobic analysts in this study. The increment of organic phase resulted in elution time and tailing factors of hydrophobic compounds decrease. From the response surface plots (Fig. 2), it was found that the appropriate analytical method with lower TF and RT for both analysts could be reach at specific mobile phase composition. Hence, the RSM was used to optimize the multiple purposes. An optimized mobile phase composition was predicted to yield RT and TF values of 12.81 min and 1.31 for adapalene, as well as 3.68 min and 1.15 for benzoyl peroxide, respectively, when level of X_1_, X_2_ and X_3_ were 25%, 50% and 25%, respectively. To validate the predictive ability of the hypothesized model for each response, a new chromatography was performed with these levels of the independent variables to yield retention time and tailing factor values of 12.09 min and 1.18 for adapalene, as well as 3.54 min and 1.15 for, benzoyl peroxide, respectively. The observed values were remarkably close to the predicted values (predicted error < 11.1%), demonstrating that the RSM could be successfully used to optimize the proportion of mobile phase.

**Table 2:** Optimal regression equation for each response variable.

	Adapalene		Benzoyl peroxide	
	TF	Ln(RT)	TF	RT
b_1_(X_1_)	1.31	1.54	1.18	2.71
b_2_(X_2_)	1.54	2.53	1.15	3.33
b_3_(X_3_)	1.11	4.22	1.08	7.11
b_12_(X_1_X_2_)	-0.31			0.05
b_13_(X_1_X_3_)	0.17			-2.93
b_23_(X_2_X_3_)	0.25			-0.99
F value	330.62	906.63	6.36	246.00
*p-value*	< 0.0001	< 0.0001	0.0266	< 0.0001
*LOF(p)*	*0*.*1701*	*0*.*1724*	*0*.*7395*	*0*.*0832*
C.V.	0.40	1.38	1.35	1.4
R^2^	0.9976	0.9962	0.6450	0.9968
Adjusted R^2^	0.9946	0.9951	0.5436	0.9927

TF: tailing factor, RT: retention time, LOF:Lack of Fit

### 3.2. Method validation

System suitability of the chromatographic analytical method was tested by estimating the repeatability, retention time, tailing factor and theoretical plates of six replicate injections of standard solution. The results listed in Table 3, the TF (1.27 and 1.12), TP (8307 and 8860), and the relative standard deviation (RSD, %) (0.69% and 0.71%) for adapalene and benzoyl peroxide, respectively, were in agreement with the recommendations of FDA [26], indicating the analytical method could provide reliable data.

**Table 3:** Results of system suitability.

Parameters	Value (Mean ±RSD %)	
	Adapalene	Benzoyl peroxide
Peak area	913465±0.69	346363±0.71
Retention time	13.4±0.35	3.82±0.12
Tailing factors	1.27±0.62	1.12±0.23
Theoretical plates	8307±0.24	8860±0.89

RSD %: Relative standard deviation

**Fig 3:**
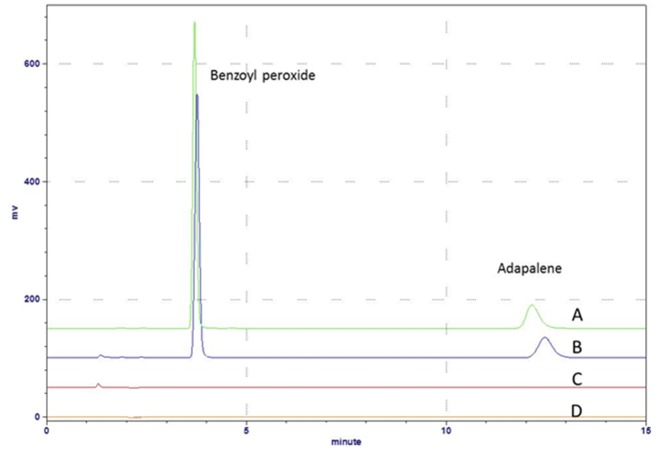
Chromatograms obtained for (A) real sample, (B) standard solution (nominal concentration), (C) blank sample, and (D) blank solution.

In term of specificity, placebo interference was carried out to investigate the specificity of the analytes (adapalene and benzoyl peroxide) in pharmaceutical preparation. The chromatograms of the blank solution, standard solution (nominal concentration), blank sample solution, and real sample solution were used to justify the specificity of the analytical method. As shown in Fig. 3, there was no interference from the other compounds, which, therefore, confirms the specificity of the method. The running time of this analytical method was 15 min, which was shorter than USP reported [18,21].

The linearity of the analytical method was determined by linear regression analysis obtaining a calibration curve by plotting the peak area versus a series of concentrations including 80%, 90%, 100%, 110% and 120% of nominal concentration of standard solution. The calibration curve obtained by analyzing adapalene was y = 32922+23913x with a correlation coefficient (r) of 0.9992, whereas the equation of a curve obtained by assaying benzoyl peroxide was y = 229017+3155x (r = 0.9996). The limits of detection of adapalene and benzoyl peroxide were 4 and 7 μg/ml, respectively. Results for accuracy, precision and recovery are presented in Table 4. All values of accuracy and precision were within acceptable limits with E% and RSD no greater than 15% [26]. The recovery ratios were greater than 97.97%. Table 5 shows the results obtained from study of robustness determined by performing small changes or variations in the method’s parameters such as detecting wavelength, flow rate and tetrahydrofuran concentration in mobile phase. None of the alterations caused a significant effect on the determination of both analytes, indicating the robustness of the developed method.

**Table 4:** Accuracy, precision and recovery of the analytes.

Theoretical Concentration (μg/ml)	Accuracy	Within-Day Variability		Between-Day Variability		Recovery
	E%	Real Concentration (mean ±S.D.)	RSD (%)	Real Concentration (mean ±S.D.)	RSD (%)	(%)
Adapalene						
32	-0.83	31.99±0.24	0.74	31.73±0.10	0.31	100.90±1.28
40	-1.63	39.59±0.36	0.90	39.35±0.46	1.17	97.97±0.32
48	-1.50	47.85±0.86	1.80	47.28±0.10	0.22	97.98±0.78
Benzoyl peroxide						
800	-0.93	795.65± 6.52	0.82	792.57± 3.72	0.47	99.02±0.01
1000	-0.72	993.52± 9.15	0.92	992.83±13.93	1.40	99.03±0.00
1200	-1.38	1192.59±18.53	1.55	1183.42± 3.22	0.27	99.02±0.01

E%: relative error of measurement, RSD (%): relative standard deviation.

**Table 5:** Robustness results of method.

	Adapalene				Benzoyl peroxide			
	Area	RT	TF	TP	Area	RT	TF	TP
Normal	906827	13.2	1.28	8562	3394717	3.8	1.134	8855
Tetrahydrofuran 26%	894564	11.9	1.28	8408	3318054	3.7	1.123	8818
Tetrahydrofuran 24%	888586	15.0	1.27	8681	3341686	4.0	1.111	9158
Detector 272 nm	834416	13.5	1.28	8818	3245174	3.9	1.132	9566
Detector 268 nm	901013	13.4	1.29	8567	3307417	3.8	1.131	9050
Flow rate 0.9 mL/mim	871002	14.1	1.30	8351	3251436	4.2	1.141	8721
Flow rate 1.1 mL/mim	858859	13.1	1.27	8461	3325463	3.7	1.132	8926

Area: peak area, RT: retention time, TF: tailing factors, TP: theoretical plates.

### 3.3. Application of the analytical method to pharmaceutical gel

In order to test the method on commercial product, the absolute recoveries of adapalene and benzoyl peroxide from commercial gel (Epiduo) containing 0.1% adapalene and 2.5% benzoyl peroxide was determined. The recoveries of adapalene and benzoyl peroxide were 93.11±0.78% and 95.17±1.02%, respectively, that fall within limits of the USP monograph.

## Conclusion

The RSM was successfully to optimize the composition of mobile phase. The selective, precise, accurate, linear and robust of this developed analytical method was met ICH guideline indicated its stability.
